# An Unusual First Presentation of Stroke and Seizure in a 32-Year-Old Patient With Brugada Syndrome Type 2 Electrocardiogram Pattern

**DOI:** 10.7759/cureus.44630

**Published:** 2023-09-04

**Authors:** John Kamara, Suresh Ponnusamy, Radim Licenik, Philip C Nwabufor, Mohmad I Rather

**Affiliations:** 1 Cardiology, Peterborough City Hospital, Peterborough, GBR; 2 Medicine, Peterborough City Hospital, Peterborough, GBR; 3 Stroke, Peterborough City Hospital, Peterborough, GBR

**Keywords:** infarct, electrocardiogram, weakness, stroke, brugada

## Abstract

We report a case of a 32-year-old lady who was admitted to the hospital with right-sided weakness that preceded an episode of seizure. On the day of admission, she woke up early in the morning with mild right-sided weakness and numbness. She had difficulty walking and later had a seizure, which was witnessed by her son. She had no signs of infection prior to this. She had no fever, chest or abdominal pain, or urinary symptoms. In the emergency department, she complained of left-sided chest tightness and heaviness, which lasted for a few minutes with associated tachycardia, electrocardiogram (ECG) was consistent with Brugada syndrome type 2. A magnetic resonant imaging (MRI) scan of her head shows a left hemispheric infarct involving the frontoparietal cortex. She was treated for an ischaemic stroke and seizure. She made a good recovery and was discharged home on secondary stroke prevention medication with community physiotherapy. She was followed up in the cardiology, genetics, and stroke outpatient clinics. The occurrence of ECG changes consistent with Brugada syndrome, stroke, and seizure in a young patient with no other risk factors for stroke is rare.

## Introduction

Brugada syndrome (BrS) is a membrane channel disorder with a characteristic electrocardiogram (ECG) ST-segment elevation in the right precordial lead. In the absence of gross structural heart disease, it carries an increased risk of sudden cardiac death (SCD) and stroke [1].

Brugada syndrome belongs to a group of diseases known as inherited primary arrhythmia syndromes. These are heterogeneous conditions, with differentiated aetiologies. Interestingly some of these entities have not been entirely described, therefore their pathophysiology is not completely understood and their definitions are evolving [2]. Most Type 2 BrS have a saddle-back pattern with at least a 2 mm J-point elevation and at least a 0.5 mm elevation of the terminal ST segment on the ECG [3]. Diagnosis may be difficult in cases of borderline or Brugada-like repolarization patterns with or without symptoms [4].

Our case highlights the importance of thoroughly assessing stroke patients for abnormal ECG changes and the rare link between Brugada syndrome and seizure.

## Case presentation

Medical history and demographics

We present a case of a 32-year-old lady who initially presented to our hospital with right-sided weakness that preceded an episode of seizure. On the day of admission, she woke up in the early hours of the morning with mild right-sided weakness and numbness. With difficulty, she walked around her house before she developed a seizure, which was witnessed by her son. On her first day in the hospital, she complained of left-sided chest tightness and heaviness, which lasted for a few minutes with associated palpitations lasting a few seconds. She had no headache, fever, neck pain or stiffness, photophobia, phonophobia, or abdominal or urinary symptom.

She has a past medical history of bronchial asthma and migraine, which were well-controlled. She also has a history of an episode of seizure three years prior to the date of presentation. Her brother and father had a diagnosis of hypertrophic cardiomyopathy. There was no history of sudden death in her family. She smoked cigarettes and drank alcohol occasionally but never used any over-the-counter or illicit drugs. She was not on any contraceptives or hormone replacement medications.

On initial examination in the emergency department, her Glasgow Coma Scale (GCS) was 15/15, pulse rate 75 beats per minute, blood pressure 112/75mmHg, respiratory rate 18 breathes per minute, oxygen saturation 98% on room air, and she was afebrile.​ She had subtle right facial asymmetry with power 3/5 in the right upper and lower limbs and reduced sensation to fine touch on the same side. Her National Institute of Health Stroke Scale (NIHSS) score was 6. She passed her swallow assessment with good oropharyngeal reflexes and sensation.

Investigations

Her full blood count, coagulation screen, urea, creatinine, electrolytes, thyroid function test, lipid profile, blood glucose, and liver function tests were all normal. She also had normal C-reactive protein (CRP) and erythrocyte sedimentation rate (ESR). An extensive blood test as part of the screening for the underlying cause of stroke in a young patient was done (Table 1).

**Table 1:** Results of the stroke in the young blood test

Test	Result	Normal range/Interpretation
Immunoglobulin G (IgG) anti-cardiolipin antibody (U/ml)	7.5	<10
Anti-nuclear antibody (ANA)	4.1	<20
Antineutrophilic cytoplasmic antibody (ANCA)	Negative	Normal
Anti-Beta-2-glycoprotein 1 IgG Abs (U/ml)	0.9	0-10
Anti-ds deoxyribonucleic acid (IU/ml)	0.8	0-10
Treponema pallidum antibody	Negative	Normal
Antibody to human immunodeficiency virus I & II	Not detected	Normal
Dilute Russell viper venom test (DRVVT) screen	0.70 No anti-phospholipid Inhibition detected	Normal
Haemoglobin F (%)	0.6	<1.9
Haemoglobin A2(%)	2.9	2.4-3.4
Sickle screen	Negative	Normal
Haemoglobin (g/L)	150	115-165
White cell count (10^ 9^/L)	8.0	4.0-11.0
Platelets (10^9^/L)	209	150-400
B12 (pg/ml)	296	197-771
Folate (ug/L)	4.9	>3.0
Brain type natriuretic peptide ( NT-pro BNP) (pg/ml)	89	<300
Lactate (mmol/L)	1.2	0.6-2.5

**Figure 1:**
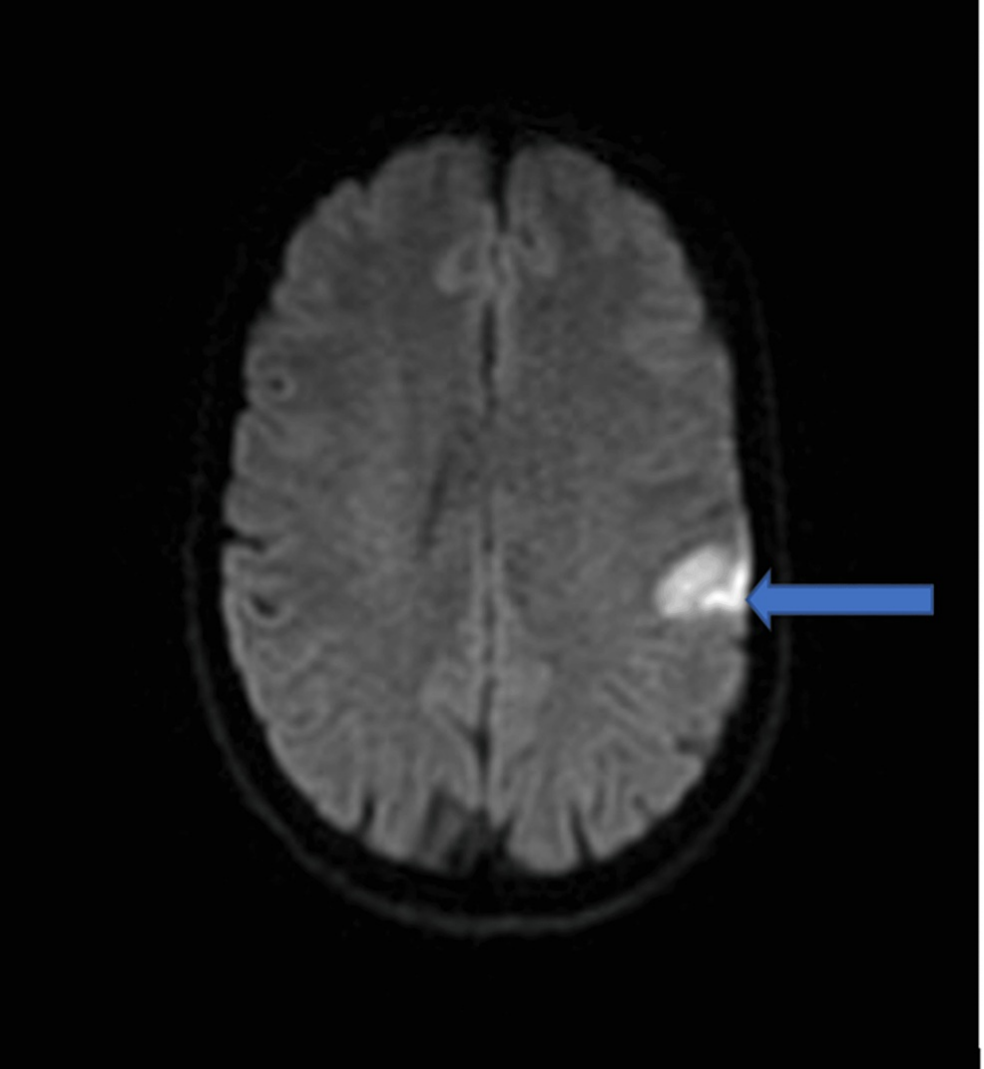
MRI DWI image showing hyperintensity in the left frontoparietal cortex (blue arrow) DWI: diffusion-weighted imaging

She also had a computed tomography (CT) scan of the brain, which did not reveal any acute intracranial pathology. We then proceeded to do a CT angiogram of the aortic arch, carotids, and intracerebral arteries, which were also normal. Following these findings, it was decided to do magnetic resonant imaging (MRI) of the brain. This revealed a left hemispheric infarct involving the frontoparietal cortex (Figure 1).

**Figure 2:**
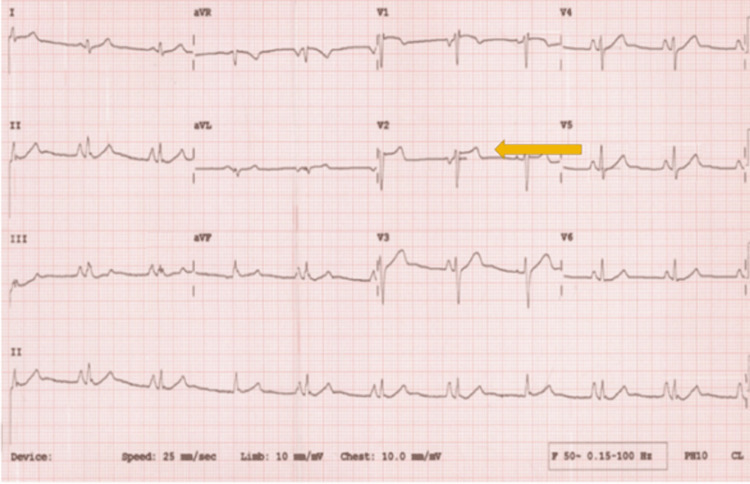
ECG showing elevated J point and ST segment changes in V1 and V2 (yellow arrow) seen in Brugada syndrome type 2

Her ECG showed elevated J point saddle ST segment changes in V1 and V2 consistent with those found in Brugada type 2 syndrome (Figure 2). A 24-hour ECG Holter was normal with no evidence of arrhythmia. A transthoracic echocardiogram was done, which showed a normal left ventricular size and function with an estimated ejection fraction of 75%, normal right ventricular size, and systolic function and valvular dysfunction. No evidence of any structural heart disease was identified. She also had a bubble echocardiogram, which was normal with no evidence of atrial or ventricular septal defects.

Treatment 

She was started on ischemic stroke secondary prevention with aspirin 300 mg once daily for two weeks followed by clopidogrel 75 mg once daily and atorvastatin 80 mg once a day. She did not receive intravenous thrombolysis as the time of onset was unknown. She was not a candidate for mechanical thrombectomy, as there was no large vessel occlusion. Levetiracetam 500 mg twice daily was initiated for early post-stroke epilepsy. During her admission, she was assessed by the physiotherapist and speech and language therapist who provided regular muscle and neuro-vascular rehabilitation.

Outcome and follow-up

At the time of discharge, she had made significant improvement in the power of her affected right upper and lower limbs. Her facial asymmetry had also improved significantly. She was advised not to drive for at least six months because of her seizure. She was followed up in the young stroke outpatient clinic. She was discharged home with secondary stroke prevention medication, including clopidogrel 75 mg once a day and atorvastatin 80 mg once a day.

She was also reviewed in the outpatient clinic two months after discharge by a consultant cardiac electrophysiologist who advised the insertion of an implantable loop recorder (ILR) to look for any significant arrhythmia. This was inserted using the sub-mammary approach. A repeat echocardiogram was organized by the cardiologist due to her family history of hypertrophic cardiomyopathy, and she will be monitored regularly by them. An outpatient appointment with a geneticist was also arranged for her.

## Discussion

Brugada syndrome belongs to a group of diseases known as inherited primary arrhythmia syndromes. It is inherited in an autosomal dominant manner. The acquired form is associated with electrolyte abnormalities such as hypercalcaemia, hypokalaemia, or hyperkalaemia, and drugs that affect cell membranes. These are heterogeneous conditions with differentiated aetiologies but with the common denominators of a genetic basis and absence of structural heart anomalies. Interestingly, some of these entities have just been recently described. Therefore, their pathophysiology needs to be wholly understood, and their definition is evolving [2].

This condition was initially described in 1992 by the Spanish cardiologists Josep and Pedro Brugada [5]. It presented a series of eight patients who displayed a right bundle branch block, a persistent ST elevation of at least 0.1 mV in the right precordial leads (V1 to V2-V3). It was the first definition of BrS, and for several years, was known as the syndrome of 'right bundle branch block, persistent ST segment elevation, and sudden death. In 1996, several reports, mainly from Japan, called the syndrome Brugada syndrome [6]. In 2002, a consensus document for the diagnosis of BrS was published. It attempted to summarize the scientific knowledge gathered since the initial description of the syndrome 10 years earlier. An operative definition of the syndrome was clearly stated [7].

There are three known ECG patterns for BrS, types 1, 2, and 3. The type 1 ECG pattern is when a prominent coved ST segment elevation displays J wave amplitude or ST-segment elevation >2 mm at its peak, followed by a negative T-wave, with little or no isoelectric separation in right precordial leads (V1 to V3). Type 2 has a high take-off of ST-segment elevation, but J wave amplitude (2 mm) gives rise to a gradually descending ST-segment elevation (remaining 1 mm above the baseline), followed by a positive or biphasic T-wave that results in a saddleback configuration; a different form of this has been observed. Type 3 is a right precordial ST-segment elevation of <1 mm of saddleback type, coved type, or both. Electrophysiology (EP) study to assess the heart's electrical activity and conduction pathways [8].

The mechanism behind most forms of inherited Brugada syndrome is not yet precise. However, in 10% to 30% of patients with this syndrome, a defect in the SCN5A gene is seen [7]. In addition, several genes are associated with Brugada syndrome, including GPD1-L, SCN1B, and SCN3B, but their role in causing it is unclear.

Brugada syndrome is associated with an increased risk of arrhythmia, including spontaneous atrial and ventricular fibrillation. This association has been demonstrated clinically and through electrophysiological tests [9]. Stroke, especially ischaemic infarcts, is linked to Brugada syndrome [10]. The incidence of cardioembolic stroke in patients with BrS and atrial fibrillation is high. Stroke is one of the main reasons for presentation in people with asymptomatic atrial fibrillation (de Asmundis et al., 2019). CHADS2 ((congestive heart failure, hypertension, age ≥75 years, diabetes mellitus, stroke (doubled)) and CHA2DS2Vasc (congestive heart failure, hypertension, age ≥75 (doubled), diabetes, stroke (doubled), vascular disease, age 65 to 74 and sex category (female)) scores did not predict the unexpectedly high risk of thromboembolic events in this group of patients [10]. People with BrS and low CHA2DDS2Vasc score are equally at risk of developing stroke. A study of 74 patients with BrS by Take et al. reported that changes of ≥0.2 mm in the ST level of the right precordial leads were more frequently observed in the VF group than in the non-VF group [11].

There is an established link between BrS and recurrent seizures as a result of cerebral involvement. The SCN5A mutation found in BrS has been linked to epilepsy, which is also a membrane channel disorder affecting the brain [12]. Autosomal dominant idiopathic epilepsies are largely due to mutations in ion channel subunits genes as well as autosomal dominant cardiac conduction abnormalities (Campuzono et al, 2011) [13]. Post-mortem studies have found mutations in the KCNH2 and SCN5A genes, all associated with arrhythmia in over 10% of people who died from a sudden unexpected death in epilepsy (SUDEP), which is a severe complication of epilepsy accounting for 18% of deaths according to Tu et al. ( 2011) [14]. This would probably explain the recurrent seizures in our patient.

There is no specific treatment for BrS; an implantable cardiac defibrillator (ICD) is inserted in patients with a high risk of cardiac death from arrhythmia or those in whom a life-threatening arrhythmia has been detected [15].

In the management of stroke, thrombolysis is offered to patients with ischaemic stroke with an NIHSS score of 6 or more. In addition, the National Institute of Health and Care Excellence (NICE) in the United Kingdom recommends alteplase as the first-line medication for thrombolysis in treating acute ischaemic stroke if given within four and half hours from the onset of symptoms and when intracranial haemorrhage has been excluded and there is no contraindication to thrombolysis [16].

Clopidogrel 75 mg once a day is recommended as the first-line antiplatelet for secondary stroke prevention. Aspirin and dipyridamole can be combined where a patient does not tolerate clopidogrel. Aspirin alone is the drug of choice when clopidogrel and dipyridamole cannot be given [16]. Patients with ischaemic stroke and atrial fibrillation or cardiac source of embolus and cerebral venous embolism should be on long-term anticoagulation unless there is a contraindication. High-intensity statins, such as atorvastatin 80 mg, once daily should be part of secondary stroke prevention. Lifestyle measures like weight reduction, healthy diet, reduction in alcohol intake, and smoking cessation should also be emphasized [17].

## Conclusions

This case serves as an important reminder to clinicians of the importance of properly screening patients presenting with stroke to look for any underlying structural, valvular, or conduction disease of the heart. Stroke is a known complication of Brugada syndrome but the co-existence of this condition with seizure in a young patient with no significant medical history is unusual. More research is needed in this area. The choice of antiplatelet in secondary stroke prevention will depend on tolerability but clopidogrel should be the first line in addition to high-intensity statins and lifestyle measures.
